# A Mouse Model of Damp-Heat Syndrome in Traditional Chinese Medicine and Its Impact on Pancreatic Tumor Growth

**DOI:** 10.3389/fonc.2022.947238

**Published:** 2022-07-25

**Authors:** Juying Jiao, Chien-shan Cheng, Panling Xu, Peiwen Yang, Linjie Ruan, Zhen Chen

**Affiliations:** ^1^ Department of Integrative Oncology, Fudan University Shanghai Cancer Center, Shanghai, China; ^2^ Department of Oncology, Shanghai Medical College, Fudan University, Shanghai, China; ^3^ Department of Chinese Integrative Medicine Oncology, The First Affiliated Hospital of Anhui Medical University, Hefei, China

**Keywords:** damp-heat syndrome, mouse model, evaluation indicators, pancreatic tumor, chemokines, cancer-associated fibroblasts

## Abstract

**Background:**

Damp-heat syndrome is one of the most important syndrome types in the traditional Chinese medicine (TCM) syndrome differentiation and treatment system, as well as the core pathogenesis of pancreatic cancer (PC) which remains a challenge to medical researchers due to its insidious onset and poor prognosis. Great attention has been given to the impact of damp-heat syndrome on tumorigenesis and progression, but less attention has been given to damp-heat modeling per se. Studying PC in a proper damp-heat syndrome animal model can recapitulate the actual pathological process and contribute to treatment strategy improvement.

**Methods:**

Here, an optimized damp-heat syndrome mouse model was established based on our prior experience. The Fibonacci method was applied to determine the maximum tolerated dosage of alcohol for mice. Damp-heat syndrome modeling with the old and new methods was performed in parallel of comparative study about general appearance, food intake, water consumption and survival. Major organs, including the liver, kidneys, lungs, pancreas, spleen, intestines and testes, were collected for histological evaluation. Complete blood counts and biochemical tests were conducted to characterize changes in blood circulation. PC cells were subcutaneously inoculated into mice with damp-heat syndrome to explore the impact of damp-heat syndrome on PC growth. Hematoxylin-eosin staining, Masson staining and immunohistochemistry were performed for pathological evaluation. A chemokine microarray was applied to screen the cytokines mediating the proliferation-promoting effects of damp-heat syndrome, and quantitative polymerase chain reaction and Western blotting were conducted for results validation.

**Results:**

The new modeling method has the advantages of mouse-friendly features, easily accessible materials, simple operation, and good stability. More importantly, a set of systematic indicators was proposed for model evaluation. The new modeling method verified the pancreatic tumor-promoting role of damp-heat syndrome. Damp-heat syndrome induced the proliferation of cancer-associated fibroblasts and promoted desmoplasia. In addition, circulating and tumor-located chemokine levels were altered by damp-heat syndrome, characterized by tumor promotion and immune suppression.

**Conclusions:**

This study established a stable and reproducible murine model of damp-heat syndrome in TCM with systematic evaluation methods. Cancer associated fibroblast-mediated desmoplasia and chemokine production contribute to the tumor-promoting effect of damp-heat syndrome on PC.

**Graphical Abstract:**
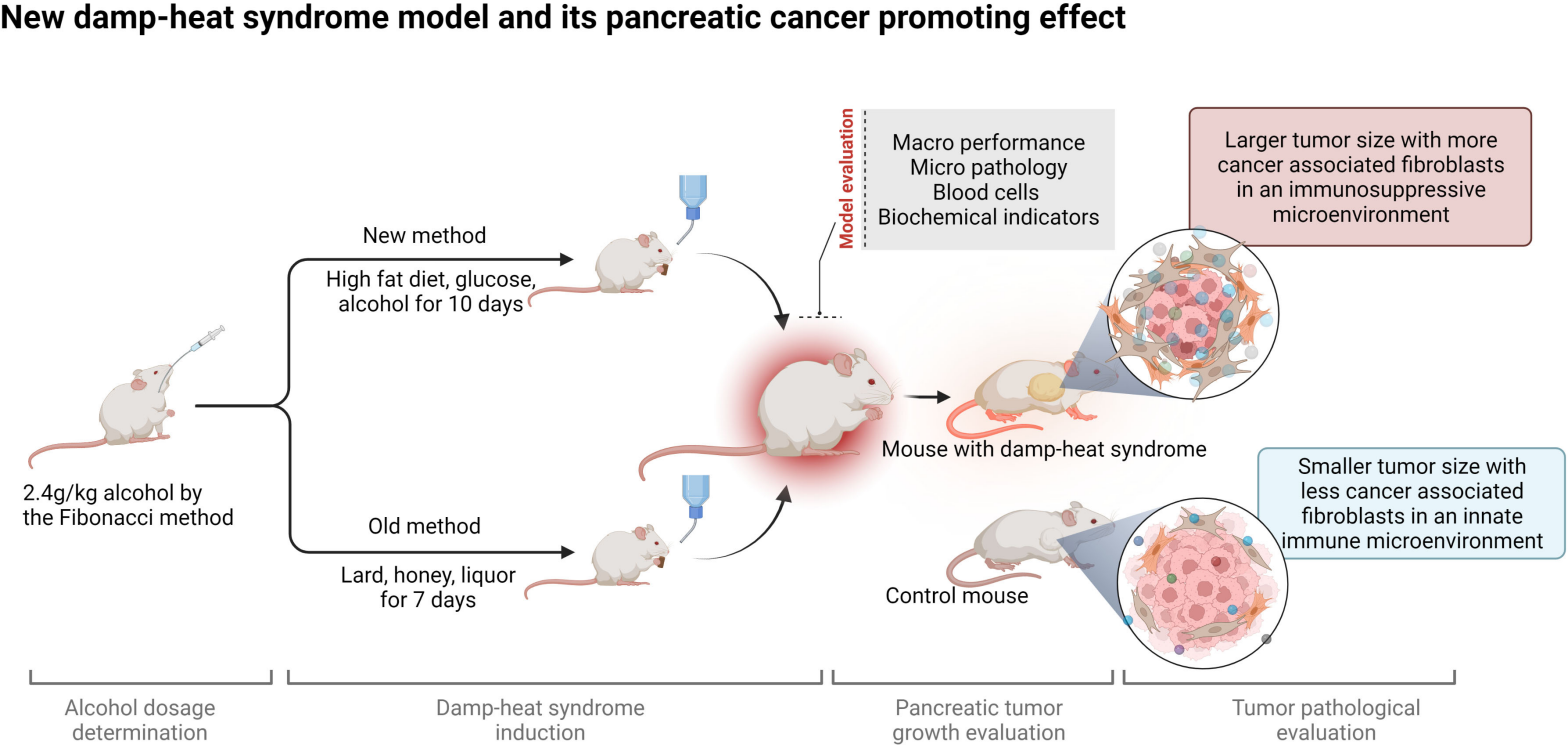


## Introduction

Damp-heat syndrome (Shi-re Zheng in Chinese) is a syndrome subtype under the traditional Chinese medicine (TCM) diagnosis and treatment system. Insights on this typical syndrome extend from conventionally febrile illness to psychosis, metabolic diseases, and various cancers (1–6); ever-increasing research has been conducted to investigate damp-heat syndrome’s essence for an evidence-based medical interpretation from the perspective of modern medicine. The historical origin, syndrome name determination, concept connotation, and main symptoms were summarized in Yuan’s review, in which the bibliometric research underscored the broad prospect of damp-heat syndrome exploration (7). Given the limitations of clinical studies in mechanism mining, an appropriate animal model with damp-heat syndrome is imperative. However, current modeling methods are heterogeneous, and widely recognized evaluation standards are lacking.

Pancreatic cancer (PC) is characterized by insidious onset and extreme malignancy. Currently, the diagnosis of PC depends primarily on the level of CA-199, and most patients have abnormal findings only when taking physical examination. Diagnosis by imaging methods, such as computed tomography and magnetic resonance imaging, can be done only when the tumor reaches a certain size. Despite many academic and theoretical reports, the early diagnosis of PC remains challenging. This unmet conundrum creates a challenge in terms of therapeutic strategies and leaves much to be desired for patients missing early surgery opportunities. On the basis of the famous saying in TCM that the outer appearance inevitably reflects the intrinsic lesions, information required for syndrome diagnosis is collected through inspection, auscultation and olfaction, inquiry, and palpation. These collected symptoms are differentiated, analyzed, and summarized as different syndromes. Accordingly, damp-heat syndrome was proposed as the core pathogenesis involved in the development and progression of PC (8). However, the unclear molecular landscape underlying damp-heat syndrome appearance hinders the widespread acceptance of syndrome-based diagnosis and treatment. Additionally, evidence-based contemporary medicine requires objective data to support the use of the TCM syndrome differentiation system.

The present study aimed to optimize methods for damp-heat modeling based on our prior experience and to propose a set of indices for model evaluation. A reproducible and stable mouse model of damp-heat syndrome was established, and the basic characteristics consisting of macro appearances, micro pathology, routine blood test parameters, and biochemical indicators, of this model were described. Studies based on this new model can enable researchers to obtain credible experimental results about the molecular basis of damp-heat syndrome, related active herbal ingredient identification, and novel strategies for disease management. Here, PC was used to study the affected phenotypes and related mechanisms when exposed to damp-heat syndrome.

## Materials and methods

### Experimental Animals

Male C57BL/6 mice aged 4-6 weeks were obtained from the experimental animal center of Fudan University Shanghai Cancer Center and housed in standard-specific pathogen-free animal barrier facilities. All experimental designs and animal operations conformed to the principles for the care and use of experimental animals. The study protocol was approved by the experimental animal ethics committee of Fudan University Shanghai Cancer Center (No. 2019JS-020).

### Determination of the Maximum Tolerated Dosage of Alcohol for Mice

**Figure 1:**
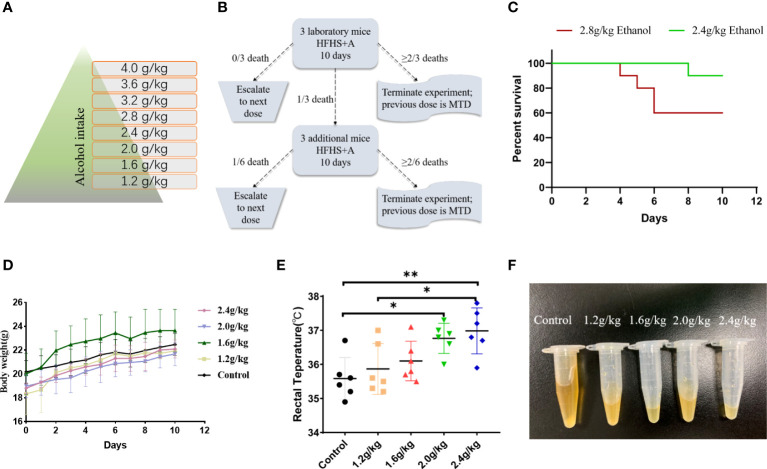
Determination of the alcohol MTD for mice. **(A)** Dose incremental scheme of alcohol. **(B)** Exploration of the alcohol MTD for mice through the Fibonacci method. **(C)** Survival of mice at the critical points and above the critical point of alcohol dosage (n = 10 in each group). **(D)** Body weight of mice receiving different dosages of alcohol (n = 10 in each group). **(E)** Rectal temperature of mice receiving different dosages of alcohol (n = 6 in each group). **(F)** Urine volume of mice receiving different dosages of alcohol (n = 6 in each group). *p < 0.05, **p < 0.01.

On the basis of previous experience that excessive alcohol intake resulted in high mouse mortality, which limited the continuation of subsequent experiments, the Fibonacci incremental method (9) was used to determine the alcohol MTD of mice. The initial absolute safe dose was set at 1.2 g/kg, which was increased by 0.4 g/kg (**Figure 1A**). The MTD of alcohol was determined according to the schematic illustration shown in **Figure 1B**. Mouse survival, body weight, and rectal temperature were recorded, and mouse urine was collected for a gross volume assessment. Intervention days were determined by considering the balance between the success rate of damp-heat syndrome modeling and the survival of mice.

### Induction of Damp-Heat Syndrome With the Old and the New Methods

To induce damp-heat syndrome, both a previous method (10) and an optimized new method were adopted. The mice in the old method group received honey water (200 g/L), a standard chow diet, and intragastric administrations of 0.3 ml dissolved lard and 0.2 ml liquor for 7 days. The mice in the new method group received glucose water (159.6 g/L), a 45% high-fat diet, and intragastric administration of 2.4 g/kg ethanol for 10 days. The mice in the control group received a standard chow diet with tap water to drink ad libitum. The general appearance, psychomotility, body temperature, food intake, water consumption, urinary volume and color, body weight, and survival status were observed and recorded. Four-time points 7, 10,14, and 35 days after induction initiation were selected for liver histological evaluation to assess the effect of damp-heat syndrome.

### Establishment of the PC Model

To explore the effect of damp-heat syndrome on PC, mice were divided into four groups: control (C group), pancreatic tumor (T group), damp-heat syndrome (D group), and pancreatic tumor with damp-heat syndrome (TD group). Mouse-derived panc02 cells were used to construct a subcutaneous PC model following damp-heat induction. In detail, 2×10^6^ cells in 200 μL PBS were injected into the right axilla of mice. The body weight of the mice was recorded every other day. The long diameter (a, mm) and short diameter (b, mm) of the formed visible tumor were measured with a Vernier caliper and recorded every three days. Tumor volume was calculated using the formula V=ab^2^/2 (mm^3^). After 3-4 weeks, the mice were euthanized, and the subcutaneous tumors were resected entirely and weighed. The abdominal fat tissue content was observed and recorded. Major organs, including the liver, kidneys, heart, spleen, lungs, and pancreas, and tumor tissue were separated and fixed in paraformaldehyde for histological evaluation. Part of the tumor tissue was cryopreserved for chemokine assays.

### Histopathological Evaluation

The fixed tissues were embedded in paraffin and sliced into 4 μm sections, and then tissue slices were dewaxed and hydrated. The preprocessed tissues were stained with hematoxylin-eosin (HE) and Masson reagents and observed under a light microscope (Leica, Wetzlar, Germany). For immunohistochemistry, tissue sections were blocked with 10% goat serum and incubated with primary antibodies at 4°C overnight. Subsequently, HRP-labeled secondary antibodies were linked, and the chromogenic substrate was added for observation. The primary antibodies used were as follows: Ki67 (1:500, CST, USA), α-SMA (1:250, CST, USA), and FAP (1: 300, Abcepta, China).

### Complete Blood Count and Biochemical Tests

Mouse blood was collected by cardiac puncture after anesthesia. A portion of the whole-blood sample was put into ethylene diamine tetraacetic acid (EDTA)-coated 1.5 ml tubes for complete blood count analysis with an automated blood analyzer (SIEMENS ADVIA 2120i, Siemens Healthcare Diagnostics Inc., Germany). The other portion of the whole-blood sample was centrifuged to obtain serum for biochemical testing with an automatic biochemical analyzer (SIEMENS ADVIA XPT, Siemens Healthcare Diagnostics Inc., Germany).

### Chemokine Microarray

A microarray containing 31 chemokines was applied to characterize the cytokine profiles in the serum and tumor tissues of mice with the Luminex multiple cytokines assay platform. Nineteen serum samples (n = 10 in the T group, n = 9 in the TD group) were diluted twofold before use. Three tumor tissues in each group were randomly selected for preliminary cytokine screening. Tissue samples were preprocessed with a lysis buffer specialized for multifactor detection (abs9225, absin, Shanghai). The concentration of the obtained total protein was adjusted to 6 μg/μL. A 96-well microplate was added to 50μL of bead mixture in each well and placed on a magnetic frame for bead adsorption. After two washes, fifty microliters of standard product and sample were added and incubated for 30 minutes with shaking at room temperature. The microplate was placed on a magnetic frame and washed three times, and then 25 μL of diluted biotin-labeled antibody complexes was added to each well and incubated for 30 minutes. Afterward, fifty microliters of diluted streptavidin-tagged PE were added for a 10-minute reaction, and the plate was washed three times. Each well was resuspended in 125 μL bead-containing assay buffer, and the plate was loaded in a Luminex 200 for detection.

### Quantitative Real-Time Polymerase Chain Reaction

The total RNA of tumor tissue was extracted using a SteadyPure Universal RNA Extraction Kit (AG21017, Accurate Biology, China). The obtained RNA was reverse transcribed into cDNA using Evo M-MLV RT Premix (AG11706, Accurate Biology, China). Quantitative RT–PCR was conducted with a SYBR Green Premix Pro Taq HS qPCR Kit (AG11718, Accurate Biology, China) on the QuantStudio 7 Flex from Applied Biosystems (ABI, USA). The 2^-ΔΔCT^ method was used to calculate the relative mRNA expression of target genes (11). The primers used are shown in **Supplementary Table S1**.

### Western Blotting

Total protein of tumor tissue samples was extracted and denatured for electrophoresis with 16.5% tricine gel (ShareBio, China). The separated proteins were transferred to PVDF membranes and then hybridized with primary and HRP-conjugated secondary antibodies (CST, America), respectively. Target protein bands were visualized with a gel imaging system (Tanon, China). The primary antibodies for IL6 (1:1000), CCL3 (1:1000), CCL4 (1:5000), CCL22 (1:3000), CXCL11 (1:2000) were purchased from Abcam (Cambridge, England); CCL2 (1:1000), CCL20 (1:500), CXCL1 (1:500), CXCL16 (1:500) were purchased from Affinity Biosciences (Jiangsu, China); CCL27 (1:500) was purchased from ABclonal Technology (Boston, USA).

### Statistical Analysis

Statistical analyses were performed by SPSS software. Student’s t-tests or one-way analysis of variance were used for the comparison of the average of two or more groups. To determine the damp-heat syndrome-related factors among the complete blood count and biochemical indicators, the absolute value of log_2_(fold change) between the model and control groups was set as more than 0.5 to screen out variables for multivariate logistic regression analyses. A p value less than 0.05 was regarded as statistically significant.

## Results

### MTD of Alcohol for Mice to Induce Damp-Heat Syndrome

A total of 50 mice were used in these experiments. Initially, ten mice were used in the 1.2 g/kg group, and 3 of them were randomly assigned as the subjects of the Fibonacci experiment. No death was observed with the initial dosage, and another 10 mice were used in the 1.6 g/kg group with 3 of them randomly assigned as the subjects of the Fibonacci experiment. The same was performed with the subsequent dosage levels (**Figure 1A**) until termination at the 2.8 g/kg dosage according to the Fibonacci method (**Figure 1B**). An ethanol dosage of 2.4 g/kg for 10 consecutive days was determined in our study to be the MTD of mice, which was defined as the optimal dosage to ensure the success rate of damp-heat syndrome induction, with greatly decreased mouse mortality. Dosages higher than 2.4 g/kg caused more deaths in 10 days (n = 10 in each group, **Figure 1C**). In addition, alcohol induction at 2.4 g/kg had no significant influence on mouse body weight (n = 10 in each group, **Figure 1D**). Although damp-heat syndrome is traditionally thought not to be accompanied by an apparent temperature increase, we observed a slight rectal temperature increase in mice given alcohol compared with control mice that received intragastric administrations of potable water (n = 6 in each group, **Figure 1E**). Additionally, a significant reduction in urine volume was observed in the mice that received alcohol gavage (n = 6 in each group, **Figure 1F**).

### Damp-Heat Syndrome Was Successfully Induced by an Optimized New Method

**Figure 2:**
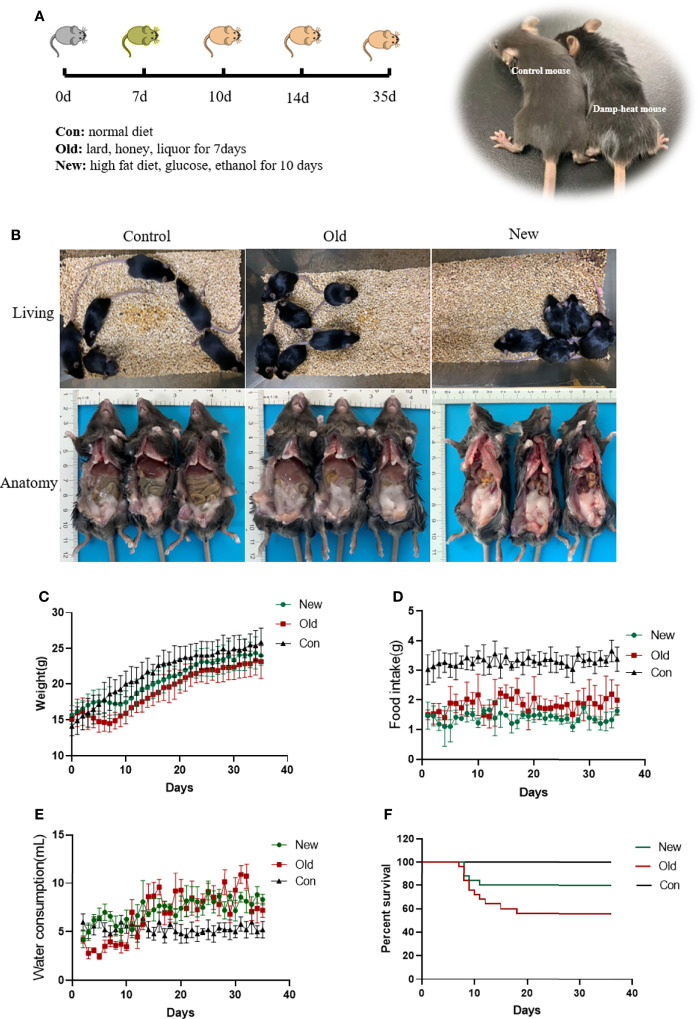
A damp-heat syndrome mouse model was established with the new and old methods. **(A)** Diagram for establishing a mouse model of damp-heat syndrome. **(B)** Activity status and anatomical view of mice in the three groups. **(C)** Body weight of mice over time. **(D)** Food intake of mice in the three groups over time. **(E)** Water consumption of mice in the three groups over time. **(F)** Survival of mice in the three groups over time.

The optimized new induction method successfully recapitulated the damp-heat syndrome appearances achieved by the previous old method (**Figure 2A**). Mice with damp-heat syndrome exhibited a propensity to flock together and increased abdominal fat content without significant body weight gain (**Figures 2B**, **C**). During the first 10 days of alcohol gavage, mice showed noticeable fluctuations in body weight and then recovered to a steady weight gain (**Figure 2C**). They also showed changes, including greasy hair, elevated skin temperature, loose stool, less urine, and smelly odor. The food intake of mice with damp-heat syndrome decreased (**Figure 2D**), while their water consumption increased (**Figure 2E**). Importantly, the new method was associated with a higher survival of mice (**Figure 2F**).

### Histological Characteristicsof Mice With Damp-Heat Syndrome

**Figure 3:**
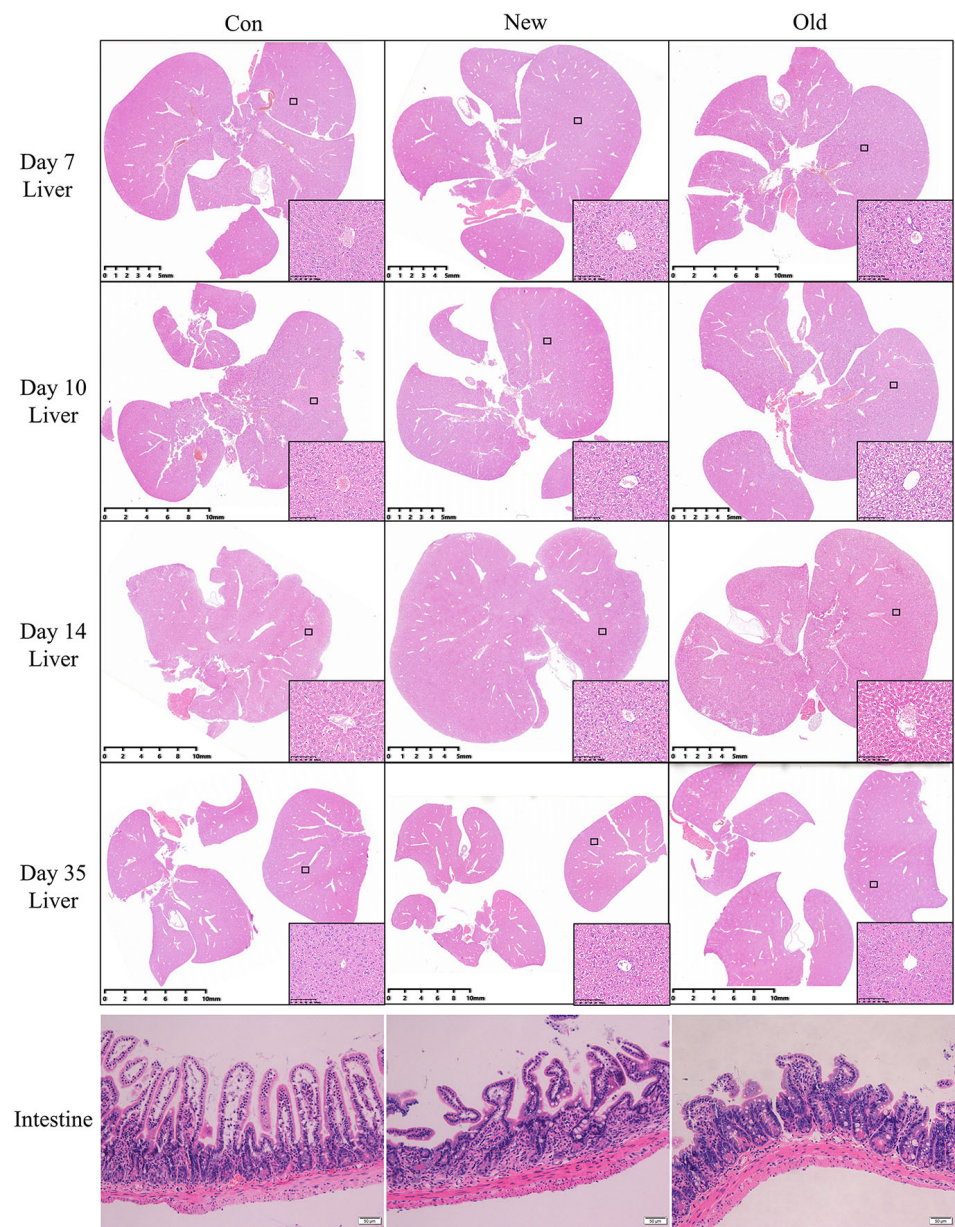
HE liver staining results of mice in the three groups at different time points after damp-heat induction and pathological observation of the intestine at the endpoint.

We observed similar histopathological changes in the major organs of the mice in the new method group and those in the old method group (**Figure 3**), suggesting that the new method can recapitulate the micro pathological changes induced by the old method in addition to the changes in external appearance. These changes were mainly located in the liver. Compared with the mice in the control group, the livers of damp-heat mice in the New and Old groups presented mild steatosis and extensive vacuolar degeneration after 7 days of induction. These pathological changes persisted on days 10, 14 and 35. In addition, compared with the control group, mice with damp-heat syndrome in the New and Old groups exhibited disarranged and shortened intestinal villi, which indicate damage to the integrity of the intestinal mucosal barrier. No significant organic lesions in the lungs (**Supplementary Figure 1**), testes (**Supplementary Figure 2**), pancreas (**Supplementary Figure 3**), spleen (**Supplementary Figure 3**), or kidneys (**Supplementary Figure 4**) were observed.

### Complete Blood Count and Biochemical Indicators of Mice With Damp-Heat Syndrome

**Figure 4:**
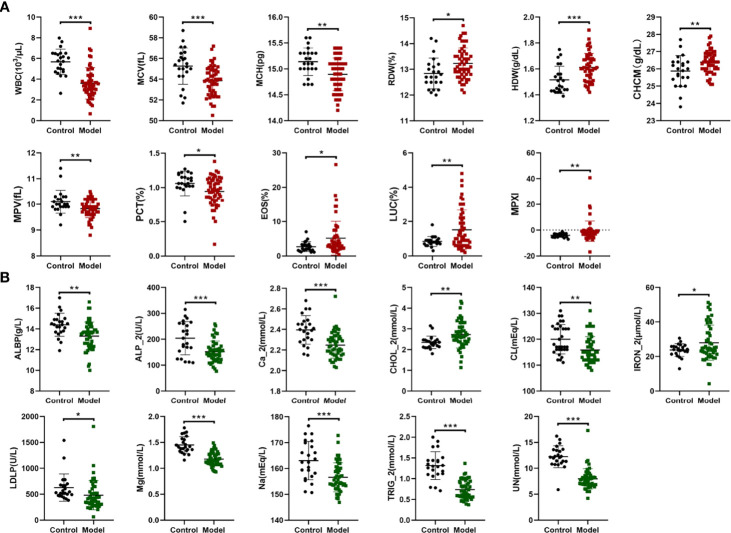
Complete blood count and biochemical analyses. **(A)** Differential complete blood count indicators between the control group and the damp-heat syndrome group. **(B)** Differential biochemical indicators between the control group and the damp-heat group. Control: healthy mice (n = 23), Model: mice with damp-heat syndrome (n = 54). *p < 0.05, **p < 0.01, ***p < 0.001.

Complete blood counts showed that the white blood cells (WBC) count, mean corpuscular volume (MCV), mean corpuscular hemoglobin (MCH), mean platelet volume (MPV), and platelet hematocrit (PCT) significantly decreased in mice in the model group when compared with the control group. In contrast, the red blood cell volume distribution width (RDW), hemoglobin content distribution width (HDW), mean hemoglobin concentration determined directly (CHCM), percentage of eosinophil cells (EOS), percentage of large unstained cells (LUC) and myeloperoxidase index (MPXI) increased significantly in mice in the model group compared with the control group (**Figure 4A** and **Supplementary Table S2**). Multivariate regression analyses indicated that the WBC count, LUC and MPXI were significantly related to damp-heat syndrome model (**Table 1**). Biochemical indicator tests showed that albumin (ALBP), alkaline phosphatase (ALP_2), triglyceride (TRIG_2), sodium (Na), chlorine (Cl), magnesium ion (MG), calcium ion (Ca_2), urea nitrogen (UN) and lactate dehydrogenase (LD) were significantly decreased in mice in the model group when compared with the control group. At the same time, total cholesterol (TC) and iron (IRON_2) increased significantly in mice in the model group compared with the control group (**Figure 4B** and **Supplementary Table S3**). Multivariate regression analyses indicated that TRIG_2 and UN were significantly related to damp-heat syndrome model (**Table 1**). Taken together, the factors WBC count, LUC, MPXI, TRIG_2 and UN can be applied as indicators of damp-heat syndrome.

**Table 1:** Multivariate regression analyses based on complete blood count and biochemical indicators for determining damp-heat related factors.

Factors	B	SE	Walds	Sig.^*^	Exp (B)
**Complete blood count**					
WBC (10^3^/μL)	-1.408	0.439	10.284	0.001	0.245
EOS (%)	0.401	0.307	1.708	0.191	1.493
LUC (%)	4.084	1.392	8.604	0.003	59.377
MPXI	0.539	0.18	8.992	0.003	1.714
**Biochemical indicators**					
TRIG_2 (mmol/L)	-5.363	1.836	8.537	0.003	0.005
UN (mmol/L)	-0.51	0.204	6.271	0.012	0.6

^*^ Logistic regression (model=1, control=0), p < 0.05 indicates a statistically significant factor.

### Impact of Damp-Heat Syndrome on PC

**Figure 5:**
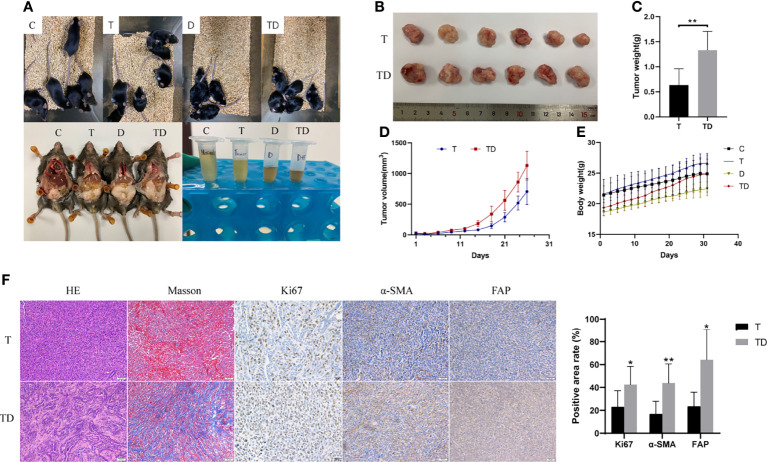
Impact of damp-heat syndrome on the pancreatic tumor. **(A)** Appearance of mice in the four groups. **(B)** Tumors of mice with and without damp-heat syndrome. **(C)** Tumor weight of mice at the end of the experiment. **(D)** Tumor volume changes over time. **(E)** Body weight of mice in the four groups. **(F)** Histopathological observation of tumor tissue. C: control group, T: pancreatic tumor group, D: damp-heat syndrome group, TD: pancreatic tumor with damp-heat syndrome group. n = 6 in each group. *p < 0.05, **p < 0.01 compared with the T group.

After the damp-heat mouse model was established with the new induction method, we first performed a repeated phenotypic validation of the effects of damp-heat syndrome on PC and continued further in-depth exploration. First, the appearance and behavior of the mice with damp-heat syndrome described above were similarly seen in pancreatic tumor-bearing mice with damp-heat syndrome (**Figure 5A**, upper panel). Consistent with the findings of our previous study (10), damp-heat syndrome significantly promoted the growth of pancreatic tumors (**Figures 5B–D**); this was further evidenced by Ki67 staining (**Figure 5F**). In addition, tumor-bearing mice with damp-heat syndrome seemed to have a more rapid body weight gain than the mice of other groups (**Figure 5E**). We observed a significant increase in abdominal fat tissue content in tumor-bearing mice with damp-heat syndrome compared with those without damp-heat syndrome (**Figure 5A**, lower left). In addition, tumor-bearing mice with damp-heat syndrome showed typical manifestations of less and yellow urine (**Figure 5A**, lower right). HE staining suggested that more abundant interstitial components appeared in the TD group than in the T group. Masson staining indicated the presence of more collagenous fibers in the TD group than in the T group. Immunohistochemistry assays showed increased expression of α-SMA and FAP in the TD group compared with the T group, indicating an increased number of fibroblasts in the tumors of mice with damp-heat syndrome.

### Chemokine Profile Changes in Tumor-Bearing Mice With Damp-Heat Syndrome

**Figure 6:**
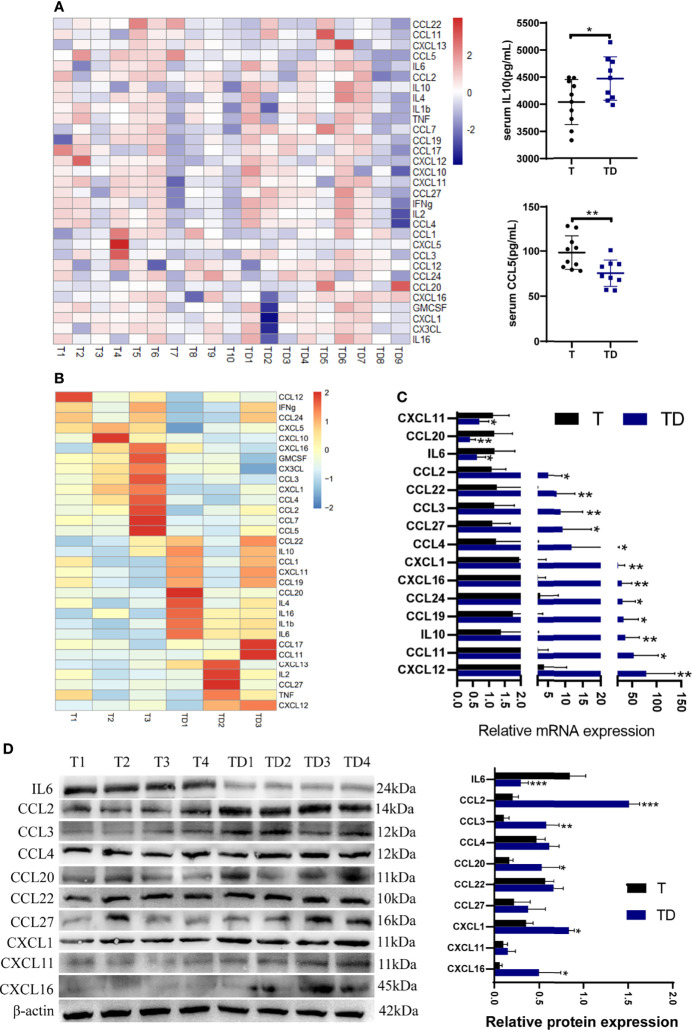
Chemokine differences between tumor-bearing mice with and without damp-heat syndrome. **(A)** Heatmap indicating serum chemokine distribution between the two groups (n = 10 in the T group, n = 9 in the TD group). **(B)** Heatmap indicating tumor tissue chemokine distribution between the two groups (n = 3 in each group). **(C)** Relative mRNA expression of tissue chemokines tested by qPCR (n = 10 in the T group, n = 9 in the TD group). **(D)** Relative protein expression of tissue chemokines tested by western blotting (n = 8 in each group). *p < 0.05, **p < 0.01, ***p < 0.001 compared with the T group.

A chemokine microarray including 31 factors was applied to explore chemokine changes in tumor-bearing mice with damp-heat syndrome. The results showed two serum chemokine differences: IL10 levels were significantly increased, while CCL5 levels were significantly decreased in the TD group (n = 9) compared with the T group (n = 10) (**Figure 6A**). Next, we evaluated the chemokine levels in the tumor tissue of mice with and without damp-heat syndrome. A preliminary screening based on the microarray suggested a tendency for a differential chemokine distribution between the T and TD groups (**Figure 6B**). The expression of these chemokines in tumor tissue was then detected at the mRNA level. PCR results showed that three chemokines, including IL6, CCL20, and CXCL11, were downregulated in the TD group compared with the T group (**Figure 6C**). Twelve chemokines, including IL10, CCL2, CCL3, CCL4, CCL11, CCL19, CCL22, CCL27, CXCL1, CXCL12, and CXCL16, were upregulated in the TD group compared with the T group (**Figure 6C**). Among these differential chemokines, IL10, CCL11, CCL19 and CXCL12 showed consistent results with the microarray detection. Considering the potential bias from the sample size for microarray test, the remaining 10 uncertain chemokines were repeatedly tested by western blotting method. The results showed that IL6 was decreased in the TD group compared with the T group, while CCL2, CCL3, CCL20, CXCL1 and CXCL16 were increased in the TD group compared with the T group (**Figure 6D**). Taken together, damp-heat syndrome induced the downregulation of IL6 and the upregulation of IL10, CCL2, CCL3, CCL11, CCL19, CCL20, CXCL1, CXCL12 and CXCL16 in the tumor tissue.

## Discussion

Through an extensive literature survey and reality-based considerations, the induction of damp-heat syndrome through a diet high in fat and containing sweet and greasy foods was selected as the appropriate method of establishment. We replaced the traditionally used lard, honey, and white spirits with a diet for which fat, glucose, and ethanol ingredients, respectively, were accurately labeled for better reproducibility. Given that alcohol use leads to high mortality in mice, the Fibonacci incremental method was applied to determine the alcohol MTD of mice. After that, with mice on a standard chow diet set as a control, the traditional old induction method and the optimized new induction method were conducted in parallel to observe whether the new method could recapitulate the general appearance of mice with damp-heat syndrome induced by the old method. Successfully, the mouse model induced by the new method not only meets the evaluation criteria previously applied to the old model but is superior in model standardization, simplicity, and reproducibility.

In addition to modeling method improvement, a systematic paradigm for model evaluation is as important as modeling. First, the fundamental manifestations of damp-heat syndrome were observed, such as damp and sticky hair, decreased food intake and increased water drinking, loose stool, less and yellow urine, and elevated skin and rectal temperature. These behaviors and appearances align with the traditional understanding of damp-heat syndrome in TCM. However, contemporary integrative medicine requires additionally quantifiable indicators to support the diagnosis of disease and syndrome. Therefore, common clinical diagnostic tests, including pathological analysis, a complete blood count, and a biochemical analysis, were used to characterize damp-heat syndrome. The pathological analysis of major organs shows that damp-heat syndrome-associated lesions were mainly located in the liver and were characterized by mild steatosis and extensive vacuolar degeneration. Liver pathological changes over time indicated an early occurrence and long-term existence of hepatic function impairment, to some extent reflecting the lingering and hard-to-heal nature of damp-heat syndrome. In addition, the disturbed intestinal villi of mice with damp-heat syndrome may explain the frequently observed gastrointestinal symptoms of patients with damp-heat syndrome in the clinic. Moreover, damp-heat syndrome leads to abnormal hematological changes mainly reflected by the white blood cell count, hemoglobin level, and the myeloperoxidase index. Biochemical abnormalities were primarily reflected by liver function-associated indicators such as albumin and alkaline phosphatase levels; dyslipidemia, such as cholesterol and triglyceride levels; and electrolyte and trace element disturbances. These observations suggest that damp-heat syndrome results in a pathophysiological state of liver dysfunction, metabolic stress, and chronic inflammation. Damp-heat syndrome evaluation based on these findings, ranging from general appearance, and gross pathology to objective blood indicators can be a favorable reference. Because current studies on damp-heat syndrome are mostly based on male subjects (7), our modeling and evaluation were also conducted with male mice. However, it is still worth noting that sex differences have been shown to affect the behavioral and pathological appearances of patients with damp-heat syndrome (7); this limitation in our study warrants further investigation. More attention should be given to sex differences when studying particular diseases, especially those susceptible to sex hormones.

In summary, the following easy-to-follow criteria were recommended for the evaluation of the establishment of a damp-heat syndrome mouse model. First, the general appearance includes sticky hair, smelly odor, elevated skin temperature, reduced food consumption, increased water intake, loose stool, less urine, activity reduction, a propensity to flock together, and abdominal fat aggregation. Second, pathological changes in the liver include mild steatosis and extensive vacuolar degeneration. Intestinal villi disarrangement can be a secondary criterion. Third, there is a decrease in the WBC count, TRIG_2, and UN, and an increase in LUC and MPXI in the blood. The damp-heat syndrome model can be thought successfully established, according to the first criterion that we are used to apply, or the objective micro indicators combining the second and third criteria. A systematic evaluation based on an integrated exterior general performances and interior micro indicators is highly recommended.

The PC model was established based on the optimized new damp-heat syndrome model to revalidate the previously observed protumor effect of damp-heat syndrome on pancreatic tumors (10). Once again, the pancreatic tumor growth-promoting role of damp-heat syndrome was confirmed, and relevant mechanisms were explored in this study. In the tumor tissue of mice with damp-heat syndrome, the proportion of the cellular interstitium increased, and collagenous fibers constituted the main noncellular composition. Cancer-associated fibroblasts (CAFs) serve as the most abundant stromal cells to produce collagen (12), and they were found to increase in tumors of mice with damp-heat syndrome. These results indicate that damp-heat syndrome promotes the proliferation of CAFs, thus leading to collagen deposition. A rich fibrotic stroma, a typical PC feature, can promote tumor progression and cause chemotherapy and immunotherapy resistance (13). Traditional herbal medicine against damp-heat syndrome could inhibit CAFs in PC (14), encourage disease control, and reverse drug resistance.

CAFs can secrete various chemokines to shape an immunosuppressive tumor microenvironment (TME) and prevent tumor cells from immune attack (15). The IL10, CCL2, CCL3, CCL11, CCL19, CCL20, CXCL1, CXCL12 and CXCL16 were increased in tumor tissues of mice after damp-heat induction, which may be partly explained by the increased CAFs activity observed in our study. IL10 functions as an immunosuppressive factor in the TME (16), and CAFs can augment IL10 secretion in PC (17). High-fat diet can induce the activation of peroxisome proliferator-activated receptor-delta which triggered CCL2 secretion by KRAS mutant pancreatic epithelial cells. CCL2 recruited suppressive macrophages and myeloid-derived cells to shape an immunosuppressive TME and promote PC development (18). Inflammatory fibroblasts can release CCL3 to recruit myeloid cells by CCR1 receptor and promote immunosuppression in PC (19). CCL11 has been reported to mediate the epithelial cell proliferation promoted by pancreatic stellate cells, which are the primary precursor cells of fibroblasts (20). Another study identified that CCL11 was primarily expressed by α-SMA+ fibroblasts in the tumor tissue of PC patients and was involved in an enhanced stromal reaction (21). CCL19 was proposed to be a double-edged sword in cancer (22). High expression of CCL19 was associated with a favorable prognosis in PC, which was attributed to its chemotactic role in immune cells (23). However, CCL19 stimulation can induce α-SMA expression and promote the epithelial-mesenchymal transition of PC cells (24), which agrees with our finding that the high CCL19 induced by damp-heat syndrome was accompanied by high α-SMA expression and increased interstitial composition. CCL20 can be released in both autocrine and paracrine ways in PC to recruit TAMs followed by cytotoxic T cells suppression and tumor progression *via* CCR6 receptor (25). CXCL1 secreted by PC cells partly determined a non-T-cell-inflamed immunosuppressive TME (26, 27). CXCL12 is secreted predominantly by FAP+ CAFs and is covalently bonded with keratin 19 to form filamentous networks in PC, thus rendering immune evasion and resistance to immune checkpoint inhibitors (28, 29). Overexpression of CXCL16 can promote KRAS-induced pancreatic tumor growth (30). Apart from the increased chemokines, IL6 was the only decreased chemokines detected in our study. The IL6 downregulation in damp-heat syndrome was consistent with our previous finding (10), but in which the low IL6 expression was contradictory to the high tumor associated macrophages (TAMs) infiltration and thus failed to explain the pancreatic tumor promoting role of TAM-derived IL6 in damp-heat syndrome. We surmised that the increased CAFs induced by damp-heat syndrome might suppress the IL6 production of TAMs and drive pancreatic tumor growth by promoting desmoplasia, this complicated intercellular interaction remains intriguing to be explored.

In addition to local changes in the tumor tissue, serum chemokine levels reflect the systematic influence of damp-heat syndrome. The increased levels of IL 10 and decreased levels of CCL5 were found in the serum of mice with damp-heat syndrome. In our previous study, PC patients with a systemic inflammation response index (SIRI) > 1.8, which indicates a poor prognosis, had a higher serum level of IL10 than those with a SIRI < 1.8. A higher IL10 level was correlated with the shortened time to progression and overall survival (31). The increased serum IL10 may originate from the tumor tissue where it was also upregulated in damp-heat syndrome. Previous studies about the role of CCL5 in PC are not inconsistent, a clinical trial suggested that low circulating levels of CCL5 predicted prolonged overall survival for PC patients receiving chemoradiotherapy (32). Another study recognized CCL5 as a helper to assist immunotherapy by mediating T-cell influx into the TME (33), accordingly, the decreased CCL5 in damp-heat syndrome seemingly lead to a systematic immunosuppression. The divergent role of CCL5 in different therapeutic strategies for PC needs to be clarified in future work. Overall, damp-heat syndrome exhibited WBC count reduction and MPXI increase, and showed circulating chemokines abnormality after re-challenge with PC, indicating some degree of immunologic derangement. Of note, chemokine alteration happened with 2 in the serum and 10 in the tumor tissue, among them, only IL10 was consistently upregulated in both serum and tumor tissue, suggesting that damp-heat syndrome affected local and systemic differently. In other words, chemokines were predominantly secreted by the tumor tissue which might be a vulnerable site to damp-heat syndrome. This at least in part underpins the core pathogenesis of damp-heat syndrome in PC. These findings demonstrated that damp-heat syndrome induces a desmoplastic niche, as well as systematic and tumor-localized immunosuppression to support pancreatic tumor growth.

## Conclusions

An optimized mouse model of damp-heat syndrome in TCM was established with the advantages of a low cost, easy operation, a stable phenotype, mouse-friendly features, and better reproducibility. This highly applicable model deserves to be popularized for research into the nature of damp-heat syndrome and the corresponding therapeutic mechanisms of Chinese herbal medicine with heat-clearing and damp-resolving efficacy. Damp-heat syndrome promotes pancreatic tumor growth, partly by triggering CAF-mediated desmoplasia and chemokine production to construct an immunosuppressive TME in PC and facilitate malignant tumor evolution.

## Data Availability Statement

The original contributions presented in the study are included in the article/**Supplementary Material**. Further inquiries can be directed to the corresponding author.

## Ethics Statement

The animal study was reviewed and approved by the experimental animal ethics committee of Fudan University Shanghai Cancer Center (No. 2019JS-020).

## Author Contributions

ZC and JJ designed the study protocol and managed the study. JJ, C-SC, PX and PY conducted the experimental work. JJ, C-SC and LR conducted the data analysis. JJ and ZC wrote and edited the paper. All authors contributed to the article and approved the submitted version.

## Funding

The work was supported by the National Natural Science Foundation of China (NSFC), under Grant [81930115].

## Conflict of Interest

The authors declare that the research was conducted in the absence of any commercial or financial relationships that could be construed as a potential conflict of interest.

## Publisher’s Note

All claims expressed in this article are solely those of the authors and do not necessarily represent those of their affiliated organizations, or those of the publisher, the editors and the reviewers. Any product that may be evaluated in this article, or claim that may be made by its manufacturer, is not guaranteed or endorsed by the publisher.

## Supplementary Material

Primers used for quantitative RT-PCR can be found in the **Supplementary Table S1**. Detailed statistical information of differential factors of complete blood count and biochemical indicators between the damp-heat syndrome model group and the control group can be found in the **Supplementary Table S2**, **S3**.

Supplementary Figure 1 Histological observation of the lung tissues of mice among the Con, New and Old groups over time.

Supplementary Figure 2 Histological observation of the testis tissues of mice among the Con, New and Old groups over time.

Supplementary Figure 3 Histological observation of the pancreas and spleen tissues of mice among the Con, New and Old groups over time.

Supplementary Figure 4 Histological observation of the kidney tissues of mice among the Con, New and Old groups over time.

## Abbreviations

ALBP, Albumin; ALP_2, Alkaline phosphatase; Ca_2, Calcium ion; CAF, Cancer associated fibroblasts; CHCM, Mean hemoglobin concentration determined directly; Cl, Chlorine; CCL, C-C motif chemokines ligand; CXCL, C-X-C motif chemokines ligand; GMCSF, Granulocyte-macrophage colony-stimulating factor; CX3CL, C-X3-C motif chemokine ligand; CCR, C-C motif chemokine receptor; EDTA, Ethylene diamine tetraacetic acid; EOS, Percentage of eosinophil cells; HE, Hematoxylin-Eosin; IL, Interleukin; INFg, Interferon gamma; IRON_2, Iron; LD, Lactate dehydrogenase; LUC, Large unstained cells; MCH, Mean corpuscular hemoglobin; MCV, Mean corpuscular volume; MG, Magnesian ion; MPV, Mean platelet volume; MPXI, Myeloperoxidase index; MTD, Maximum tolerated dosage; Na, Sodium; PC, Pancreatic cancer; PC, Platelet hematocrit; qRT-PCR, Quantitative real-time polymerase chain reaction; RDW, Red blood cell volume distribution width; SIRI, Systemic inflammation response index; TAM, Tumor associated macrophage; TME, Tumor microenvironment; TNF, Tumor necrosis factor; TC, Total cholesterol; TCM, Traditional Chinese medicine; TRIG_2, Triglyceride; UN, Urea nitrogen; WBC, White blood cell.
