# Supplementary figures and images for: A Mouse Model of Damp-Heat Syndrome in Traditional Chinese Medicine and Its Impact on Pancreatic Tumor Growth
Source: Front Oncol. 2022 Jul 25;12:947238. doi: 10.3389/fonc.2022.947238 (PMC9357947; doi:10.3389/fonc.2022.947238)

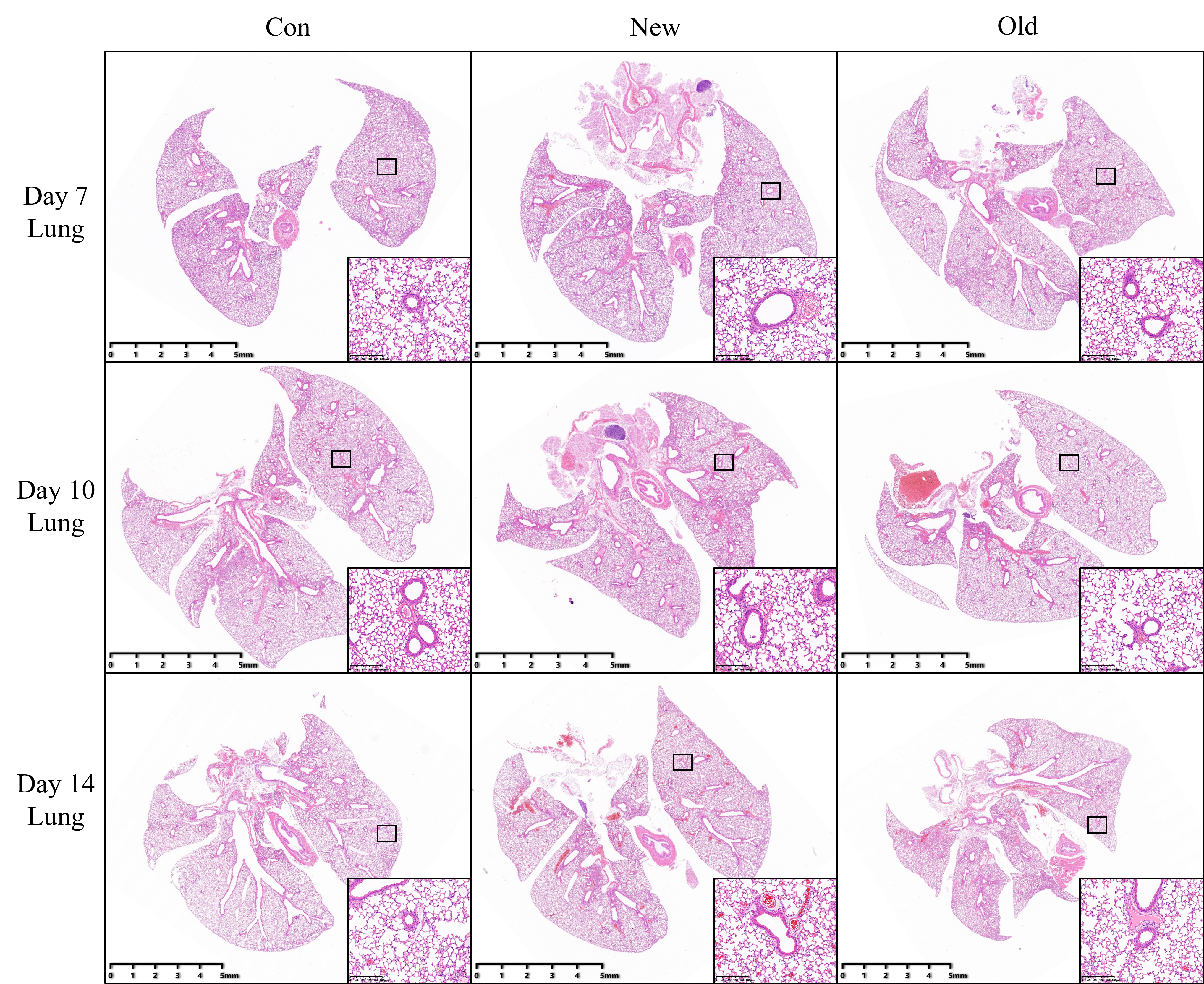

Supplement: Supplementary Figure 1 — Histological observation of the lung tissues of mice among the Con, New and Old groups over time. [file Image_1.tif]

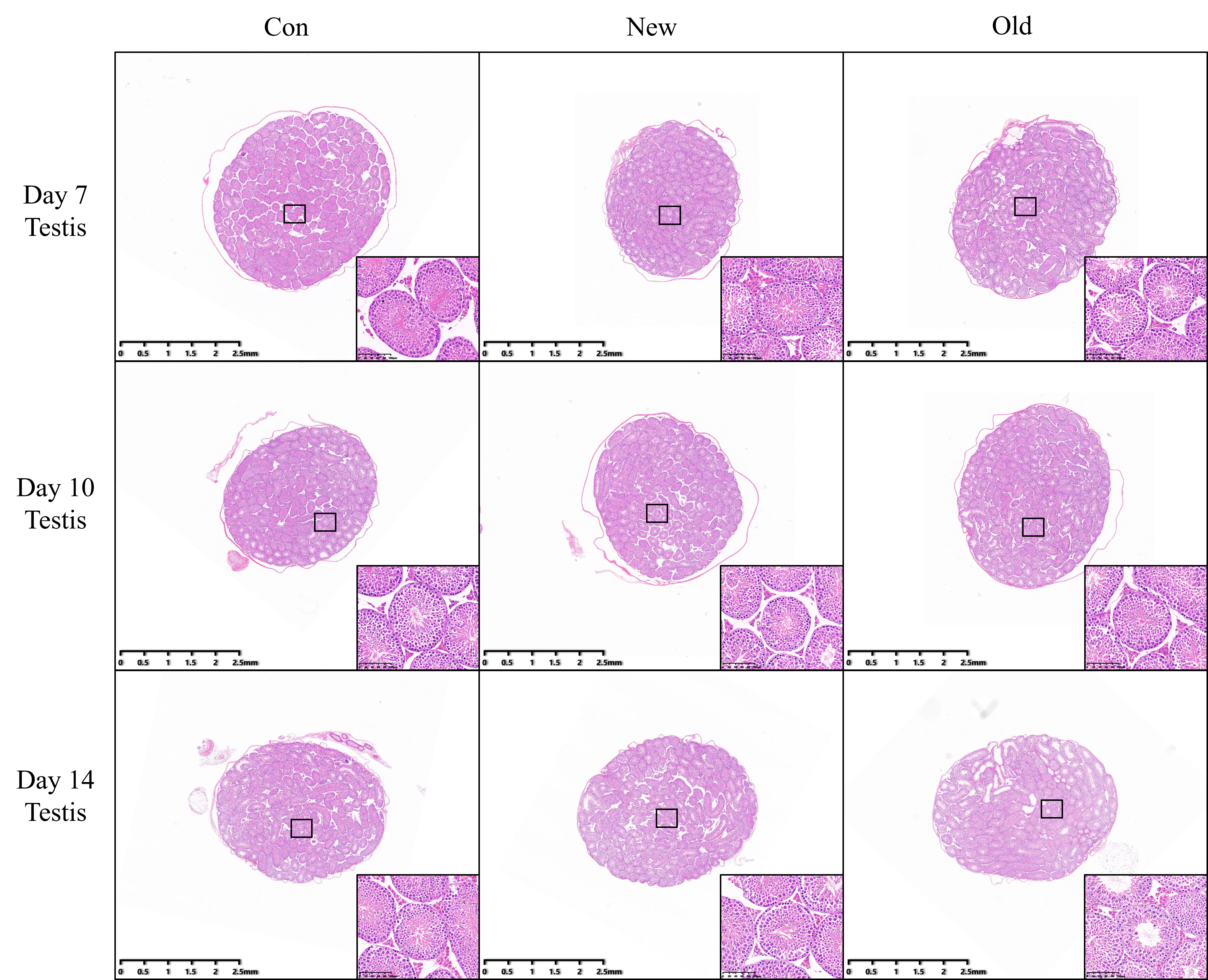

Supplement: Supplementary Figure 2 — Histological observation of the testis tissues of mice among the Con, New and Old groups over time. [file Image_2.tif]

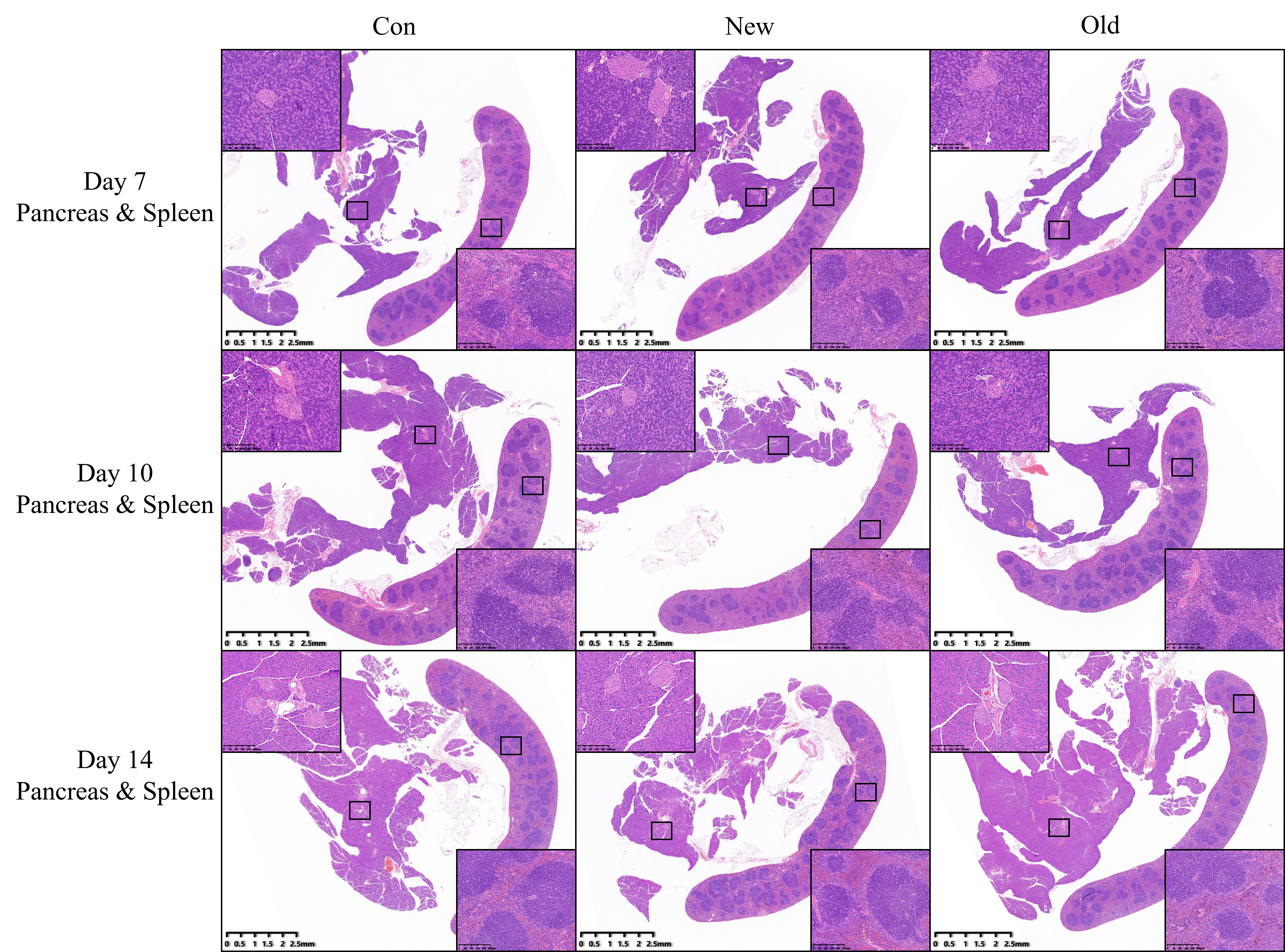

Supplement: Supplementary Figure 3 — Histological observation of the pancreas and spleen tissues of mice among the Con, New and Old groups over time. [file Image_3.tif]

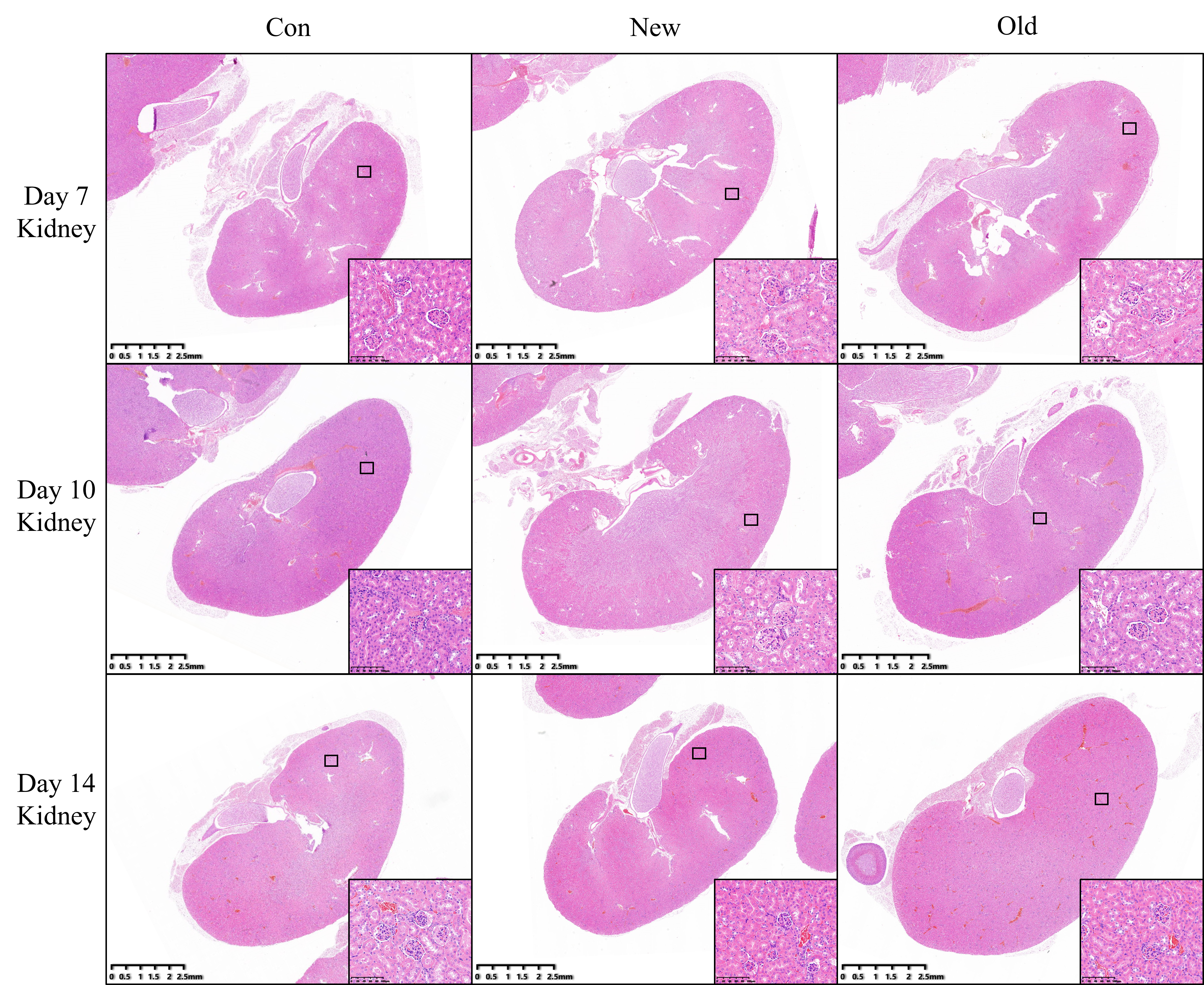

Supplement: Supplementary Figure 4 — Histological observation of the kidney tissues of mice among the Con, New and Old groups over time. [file Image_4.tif]
