# Supplementary material for: A Mouse Model of Damp-Heat Syndrome in Traditional Chinese Medicine and Its Impact on Pancreatic Tumor Growth
Source: Front Oncol. 2022 Jul 25;12:947238. doi: 10.3389/fonc.2022.947238 (PMC9357947; doi:10.3389/fonc.2022.947238)
Supplement: Supplementary file 5 [file DataSheet_1.docx]

Supplementary Material

# Table S1. Primers used for quantitative RT-PCR.

| Genes | Primer sequences |
| --- | --- |
| CCL20-F | GCCTCTCGTACATACAGACGC |
| CCL20-R | CCAGTTCTGCTTTGGATCAGC |
| IL1b-F | GCAACTGTTCCTGAACTCAACT |
| IL1b-R | ATCTTTTGGGGTCCGTCAACT |
| CXCL5-F | TGCGTTGTGTTTGCTTAACCG |
| CXCL5-R | CTTCCACCGTAGGGCACTG |
| TNF-alpha-F | CCCTCACACTCAGATCATCTTCT |
| TNF-alpha-R | GCTACGACGTGGGCTACAG |
| GM-CSF-F | GGCCTTGGAAGCATGTAGAGG |
| GM-CSF-R | GGAGAACTCGTTAGAGACGACTT |
| CX3CL-F | ACGAAATGCGAAATCATGTGC |
| CX3CL-R | CTGTGTCGTCTCCAGGACAA |
| CCL1-F | GAATCACCAACAACAGATGCAC |
| CCL1-R | ATCCTGGACCCACTTCTTCTT |
| CXCL13-F | GGCCACGGTATTCTGGAAGC |
| CXCL13-R | GGGCGTAACTTGAATCCGATCTA |
| IL-16-F | AAGAGCCGGAAATCCACGAAA |
| IL-16-R | GTCTCAAAAGGGTCAGGGTACT |
| IFN-g-F | ATGAACGCTACACACTGCATC |
| IFN-g-R | CCATCCTTTTGCCAGTTCCTC |
| CXCL10-F | CCAAGTGCTGCCGTCATTTTC |
| CXCL10-R | GGCTCGCAGGGATGATTTCAA |
| IL-2-F | GTGCTCCTTGTCAACAGCG |
| IL-2-R | GGGGAGTTTCAGGTTCCTGTA |
| CXCL11-F | GGCTTCCTTATGTTCAAACAGGG |
| CXCL11-R | GCCGTTACTCGGGTAAATTACA |
| IL-6-F | CCAAGAGGTGAGTGCTTCCC |
| IL-6-R | CTGTTGTTCAGACTCTCTCCCT |
| IL-4-F | GGTCTCAACCCCCAGCTAGT |
| IL-4-R | GCCGATGATCTCTCTCAAGTGAT |
| CCL7-F | GCTGCTTTCAGCATCCAAGTG |
| CCL7-R | CCAGGGACACCGACTACTG |
| CCL12-F | ATTTCCACACTTCTATGCCTCCT |
| CCL12-R | ATCCAGTATGGTCCTGAAGATCA |
| CCL2-F | TTAAAAACCTGGATCGGAACCAA |
| CCL2-R | GCATTAGCTTCAGATTTACGGGT |
| CCL22-F | AGGTCCCTATGGTGCCAATGT |
| CCL22-R | CGGCAGGATTTTGAGGTCCA |
| CCL5-F | GCTGCTTTGCCTACCTCTCC |
| CCL5-R | TCGAGTGACAAACACGACTGC |
| IL-10-F | GCTCTTACTGACTGGCATGAG |
| IL-10-R | CGCAGCTCTAGGAGCATGTG |
| CXCL1-F | CTGGGATTCACCTCAAGAACATC |
| CXCL1-R | CAGGGTCAAGGCAAGCCTC |
| CCL27-F | AGGAGGATTGTCCACATGGAA |
| CCL27-R | CTTGGCGTTCTAACCACCGA |
| CCL17-F | TACCATGAGGTCACTTCAGATGC |
| CCL17-R | GCACTCTCGGCCTACATTGG |
| CCL19-F | GGGGTGCTAATGATGCGGAA |
| CCL19-R | CCTTAGTGTGGTGAACACAACA |
| CXCL16-F | CCTTGTCTCTTGCGTTCTTCC |
| CXCL16-R | TCCAAAGTACCCTGCGGTATC |
| CXCL12-F | TGCATCAGTGACGGTAAACCA |
| CXCL12-R | TTCTTCAGCCGTGCAACAATC |
| CCL24-F | TCTTGCTGCACGTCCTTTATT |
| CCL24-R | GCATCCAGTTTTTGTATGTGCC |
| CCL11-F | GAATCACCAACAACAGATGCAC |
| CCL11-R | ATCCTGGACCCACTTCTTCTT |
| CCL4-F | TTCCTGCTGTTTCTCTTACACCT |
| CCL4-R | CTGTCTGCCTCTTTTGGTCAG |
| CCL3-F | TTCTCTGTACCATGACACTCTGC |
| CCL3-R | CGTGGAATCTTCCGGCTGTAG |
| GAPDH-F | GGGAGCCAAAAGGGTCATCATCTC |
| GAPDH-R | CCATGCCAGTGAGCTTCCCGTTC |

# **Table S2.** Differential factors of complete blood count between the damp-heat syndrome model and the control groups.

| Factors | Control (n=23) | Model (n=54) | t-test | *p*-value | Log_2_(FC)^*^ |
| --- | --- | --- | --- | --- | --- |
| WBC (10^3^/μL) | 5.68±1.22 | 3.6±1.51 | 5.83 | <0.0001 | -0.66 |
| MCV (fL) | 55.27±1.75 | 53.86±1.41 | 3.73 | 0.0004 | -0.04 |
| MCH (pg) | 15.14±0.27 | 14.89±0.31 | 3.27 | 0.0016 | -0.02 |
| RDW (%) | 12.84±0.6 | 13.23±0.61 | -2.59 | 0.0116 | 0.04 |
| HDW (g/dL) | 1.52±0.11 | 1.61±0.11 | -3.58 | 0.0006 | 0.09 |
| CHCM (g/dL) | 25.87±0.9 | 26.39±0.64 | -2.82 | 0.0061 | 0.03 |
| MPV (fL) | 10.1±0.45 | 9.83±0.34 | 2.92 | 0.0046 | -0.04 |
| PCT (%) | 1.06±0.18 | 0.94±0.21 | 2.3 | 0.0243 | -0.17 |
| EOS (%) | 2.68±1.43 | 5.15±4.91 | -3.38 | 0.0012 | 0.94 |
| LUC (%) | 0.84±0.28 | 1.51±1.15 | -4.01 | 0.0002 | 0.84 |
| MPXI | -4.1±1.44 | -0.91±7.88 | -2.87 | 0.0057 | -2.17 |

^*^ Log_2_(fold change), Model vs. Control.

# **Table S3.** Differential biochemical indicators between the damp-heat syndrome model and the control groups.

| Factors | Control (n=23) | Model (n=54) | t-test | *p*-value | Log_2_(FC)^*^ |
| --- | --- | --- | --- | --- | --- |
| ALBP (g/L) | 14.4±1.11 | 13.3±1.37 | 3.35 | 0.0013 | -0.11 |
| ALP_2c(U/L) | 204.03±64.16 | 151.86±41.16 | 3.58 | 0.0012 | -0.43 |
| Ca_2(mmol/L) | 2.4±0.14 | 2.25±0.13 | 4.51 | <0.0001 | -0.09 |
| CHOL_2(mmol/L) | 2.35±0.3 | 2.73±0.65 | -3.39 | 0.0012 | 0.21 |
| CL (mEq/L) | 119.35±5.84 | 115.86±5.2 | 2.56 | 0.0126 | -0.04 |
| IRON_2 (μmol/L) | 23.76±3.68 | 27.99±10.18 | -2.6 | 0.0115 | 0.23 |
| LDLP (U/L) | 627.23±263.57 | 480.66±281.3 | 2.1 | 0.0391 | -0.38 |
| Mg (mmol/L) | 1.45±0.16 | 1.18±0.13 | 7.64 | <0.0001 | -0.3 |
| Na (mEq/L) | 163.05±7.39 | 1.18±0.13 | 4.09 | 0.0001 | -0.06 |
| TRIG_2 (mmol/L) | 1.32±0.34 | 0.74±0.23 | 8.65 | <0.0001 | -0.84 |
| UN (mmol/L) | 12.25±2.12 | 7.96±1.97 | 8.44 | <0.0001 | -0.62 |

^*^ Log_2_(fold change), Model vs. Control.
